# Facile Synthesis of 1T-Phase MoS_2_ Nanosheets on N-Doped Carbon Nanotubes towards Highly Efficient Hydrogen Evolution

**DOI:** 10.3390/nano11123273

**Published:** 2021-12-02

**Authors:** Kunjie Wang, Jiahui Zhang, Yachen Ye, Hongbin Ma, Bingxin Liu, Peng Zhang, Benhua Xu

**Affiliations:** 1 Qinghai Provincial Key Laboratory of New Light Alloys, Qinghai Provincial Engineering Research Center of High-Performance Light Metal Alloys and Forming, Qinghai University, Xining 810016, China; wkjfxb@163.com (K.W.); z276475670@126.com (J.Z.); yyc5819@163.com (Y.Y.); mahb12@163.com (H.M.); liubx408@nenu.edu.cn (B.L.); 2 Chemical Engineering College, Qinghai University, Xining 810016, China

**Keywords:** 1T-MoS_2_ nanosheets, N-doped carbon nanotubes, hydrogen evolution reaction, water splitting, synergy effects

## Abstract

1T-phase molybdenum disulfide is supposed to be one of the non-precious metal-based electrocatalysts for the hydrogen evolution reaction with the highest potential. Herein, 1T-MoS_2_ nanosheets were anchored on N-doped carbon nanotubes by a simple hydrothermal process with the assistance of urea promotion transition of the 1T phase. Based on the 1T-MoS_2_ nanosheets anchored on the N-doped carbon nanotubes structures, 1T-MoS_2_ nanosheets can be said to have highly exposed active sites from edges and the basal plane, and the dopant N in carbon nanotubes can promote electron transfer between N-doped carbon nanotubes and 1T-MoS_2_ nanosheets. With the synergistic effects of this structure, the excellent 1T-MoS_2_/ N-doped carbon nanotubes catalyst has a small overpotential of 150 mV at 10 mA cm^−2^, a relatively low Tafel slope of 63 mV dec^−1^, and superior stability. This work proposes a new strategy to design high-performance hydrogen evolution reaction catalysts.

## 1. Introduction

The worsening environmental pollution consequences resulting from increasing demands of using traditional fossil fuels have encouraged humans to develop clean and renewable energy technologies to gradually replace the traditional fossil fuels [1,2,3]. Hydrogen has been considered as a potential alternative to fossil fuels because of its clean and pollution-free nature. Among the various H_2_ production technologies, electrochemical water splitting has been taken as a promising, sustainable way to produce hydrogen [4,5,6]. However, the low efficiency of hydrogen production in the hydrogen evolution reaction (HER) hinders large commercial applications [7]. Hence, various electrochemical catalysts have been developed to solve the above problems. Although scarce noble metals such as Pt and its composites have been considered the most efficient electrochemical catalysts for HER, further commercial applications are limited by their expensive price and scarcity [8,9,10,11]. Therefore, developing high catalytic performance and earth-abundant catalysts as alternatives to Pt-based catalysts for HER is crucial for realizing hydrogen production more efficiently [12,13]. At present, a large number of earth-abundant catalysts with high catalytic performance have been explored, including carbon-based materials, metal carbides, metal nitrides, metal phosphides, two-dimensional (2D) transition metal dichalcogenides (TMDs), etc. [14,15,16]. Among them, molybdenum disulfide (MoS_2_), as the representative of 2D TMDs, has been researched as a promising candidate for HER catalysis because of its efficient catalytic hydrogen activities [17]. Previous studies have shown that semiconducting 2H MoS_2_ has poor conductivity and the active sites for hydrogen absorption are limited to edge sites, whilst metallic 1T MoS_2_ has better conductivity and more active sites on both the basal plane and edges, which indicates that 1T-phase MoS_2_ possesses better catalytic hydrogen evolution activity compared with 2H-phase MoS_2_ [18]. Therefore, diverse “top-down” and “bottom-up” routes are explored to prepare 1T-phase MoS_2_. The 1T-phase MoS_2_ nanosheets are usually obtained by “top-down” methods, including chemical/electrochemical intercalation with alkali metal ions, electron beam irradiation, and plasmonic hot injection [19,20,21,22,23]. Furthermore, 1T-phase MoS_2_ nanostructures such as nanoflower, nanosheets, quantum dots, etc. [24,25,26,27], have been synthesized by “bottom-up” methods, such as hydrothermal/solvothermal methods [28]. The obtained 1T-phase MoS_2_ catalysts exhibit improved electrocatalytic hydrogen activity. To implement the practical application of 1T-phase MoS_2_ catalysts in HER, great efforts have been put into constantly improving the HER activity of 1T-phase MoS_2_ through improving conductivity and increasing active sites.

The combination of MoS_2_ with special nanostructures and conductive materials, such as graphene, carbon nanotubes (CNTs), carbon fibers, and carbon paper [10,13,29], is one of the effective strategies to improve the HER activity of 1T-phase MoS_2_. When 1T-MoS_2_ nanosheets, nanoparticles, or other nanostructures are grown on carbon supports, abundant active sites on both the basal plane and edges can be preferentially exposed and increase the electron conduction rate during the HER process. Furthermore, the conductivity and electrocatalytic activity of these carbon supports can be tuned by doping metal-free heteroatoms such as N [18,30,31]. The theoretical calculations indicate that introducing nitrogen into carbon supports could enhance the catalytic activity and the electron transport at the MoS_2+x_/N-doped carbon nanotubes (NCNTs) interface and improve catalyst adhesion and charge transfer kinetics [32,33]. Based on the above discussion, growing 1T-MoS_2_ nanosheets on doped carbon supports could enhance the electrocatalytic activity of 1T-MoS_2_-based catalysts by simultaneously improving the conductivity and preferentially exposing active sites.

Inspired by the above discussion, we prepared 1T-MoS_2_/NCNTs composites by growing 1T-MoS_2_ nanosheets on N-doped carbon nanotubes (NCNTs). The NCNTs ensured good electronic conductivity between the NCNTs and 1T-MoS_2_ nanosheets. Furthermore, the 1T-MoS_2_ nanosheets were decorated on the surface of the NCNTs uniformly, which could expose sufficient active sites and bring the active sites into full contact with electrolytes. Thus, this configuration can make 1T-MoS_2_ nanosheets accept external electrons and ensures a highly efficient catalytic reaction. The composites of 1T-MoS_2_/NCNTs showed excellent HER performance, including a low overpotential of 150 mV at a current density of 10 mA cm^−2^, low Tafel slope of 63 mV dec^−1^, and great stability after 10 h.

## 2. Experimental Section

### 2.1. Materials Preparation

CNTs were purchased from Shenzhen Nanotech Port Co. Ltd., Shanghai, China. All chemical reagents were analytical-grade and directly used without further refinement. Thioacetamide (TAA), molybdenum trioxide (MoO_3_), and urea (CH_4_N_2_O) were purchased from Aladdin Industrial Corporation, Shanghai, China.

### 2.2. Preparation of NCNTs

The CNTs were cleaned with ultrasonic washer in ethyl alcohol and with deionized water (DW) for 10 min. Then, centrifugal cleaning was applied at 5000 rpm three times using ethyl alcohol and DW, and the black powder was then dried in a vacuum oven at 60 °C for 6 h. Next, urea and CNTs powder were put in a crucible sealed with copper foil containing a porous partition in the middle and annealed at 800 °C with Ar atmosphere protection. After cooling down to room temperature, the black powder was cleaned by centrifugation at 5000 rpm three times using ethyl alcohol and DW to remove the residuals. Then, the black powder was dried in a vacuum drying oven at 60 °C for 6 h.

### 2.3. Preparation of the Catalyst

The 1T-MoS_2_/NCNTs nanocomposites were fabricated using a simple one-pot solvothermal method. Typically, 100 mg urea, 50 mg MoO_3,_ and 100 mg thioacetamide (TAA) were dissolved into a 60-milliliter mixture solution (*V*_dw_:*V*_ethyl alcohol_ = 1:1) with mechanical stirring for 30 min. After 6 mg NCNTs black powder was added into the above homogeneous solution and sonicated for 30 min, the obtained homogeneous solution was transferred to a 100-milliliter Teflon-lined autoclave and maintained at 200 °C for 18 h. After the Teflon-lined autoclave cooled down to room temperature, the black powder of the 1T-MoS_2_/NCNTs was successfully obtained after centrifugal cleaning at 5000 rpm several times using ethyl alcohol and DW to remove the residuals. The final products were dried in a vacuum oven at 60 °C for 12 h. The 1T-MoS_2_/CNTs composite was synthesized using the same reaction procedures with the addition of not NCNTs powder but CNTs powder. The 1T-MoS_2_ nanoflower products were synthesized without adding carbon nanotubes, only MoO_3_, TAA, and urea.

### 2.4. Characterization

The microstructure and morphology of the as-prepared products were observed using a scanning electron microscope (SEM, JSM-7900F, JEOL, Tokyo, Japan) and a transmission electron microscope (TEM, JEM-2100F, JEOL, Tokyo, Japan). X-ray diffraction (XRD) patterns were collected on an X-ray diffractometer (D/MAX 2500PC, Tokyo, Japan), using Cu/Kα radiation (40 kV, 200 mA). X-ray photoelectron spectroscopy (XPS) measurements were conducted on an ESCALAB X spectrometer (Waltham, MA, USA) with a monochromatic Al/Kα source (1486.6 eV). All XPS spectra were calibrated according to the C 1s peak (284.8 eV) of adventitious carbon on the analyzed sample surface. The Raman spectra were collected on a Horiba LabRAM Odyssey Raman microscope (Lille, France) using a 532 nm excitation laser.

### 2.5. Electrochemical Measurement

To prepare the working electrode, 3 mg of the synthesized black powder was dissolved into a mixed solution including 290 mL deionized water and 700 mL ethanol, and then, 10 μL Nafion solution (5 wt%) was added dropwise into the above solution to form homogenous ink by sonicating for 1 h. Then, the as-prepared ink (5 μL) was dropped uniformly onto a 3 mm diameter glassy carbon electrode and left to air-dry naturally.

The electrochemical performance was tested using a CHI 760E electrochemical workstation with a standard three-electrode system. The as-prepared glass carbon electrode, a saturated calomel electrode (SCE), and a graphite rod were employed as the working electrode, reference electrode, and counter electrode, respectively. In all measurements, the SCE reference electrode was calibrated with reversible hydrogen electrode (RHE), where E(RHE) = E(SCE) + 0.244 V in 0.5 M H_2_SO_4_ solution. Before electrochemical testing, high-purity argon was applied to purify the 0.5 M H_2_SO_4_ solution by eliminating oxygen interference. Linear sweep voltammetry (LSV) measurements with 90% manual IR compensation were taken at a scan rate of 2 mV/s from −0.1 to −0.6 V. Tafel slopes were derived from the LSV curves by fitting the following equation: *η* = *a* + *b* × log *j*. The double-layer capacitances (*C*_dll_) were derived from cyclic voltammetry (CV), tested at 0.1 to 0.2 V vs. RHE at different scan rates of 10, 20, 30, 40, and 50 mV/s. Electrochemical impedance spectroscopy (EIS) measurements were conducted at frequencies ranging from 100 kHz to 0.01 Hz with an amplitude of 5 mV. The electrochemical stability was tested by cyclic voltammetry testing for 1000 cycles and chronoamperometry at a constant voltage of 150 mV vs. RHE. All potentials in the demonstrated figures were calibrated to RHE according to the equation E(RHE) = E(SCE) + 0.244 V.

## 3. Results and Discussion

**Figure 1:**
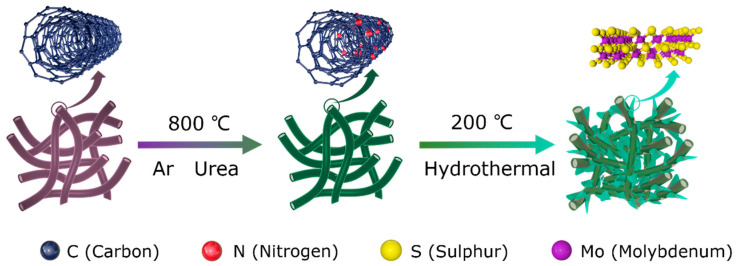
Schematic diagram illustrating the hydrothermal preparation of 1T-MoS_2_/NCNTs composite.

The overall synthesis approach of the 1T-MoS_2_/NCNTs nanostructure is shown schematically in Figure 1. First, NCNTs were fabricated from CNTs. The urea and CNTs powders were put into a crucible containing a porous partition in the middle and annealed at 800 °C with Ar atmosphere protection. A solution consisting of MoO_3_, TAA, and urea precursors dispersed in ethanol and deionized water was used to fabricate 1T-MoS_2_/NCNTs. As a result, 1T-MoS_2_ nanosheets anchored on NCNTs composites were prepared by a simple solvothermal method.

**Figure 2:**
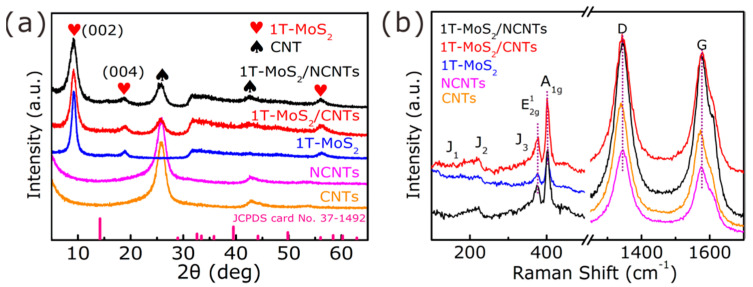
(**a**) XRD patterns of as-prepared samples. (**b**) Raman spectra of as-prepared samples.

The crystalline structure of the as-fabricated products was characterized by XRD. Figure 2a shows the XRD patterns of the CNTs, NCNTs, 1T-MoS_2_, 1T-MoS_2_/NCNTs, and 1T-MoS_2_/CNTs. It can be observed that the CNTs and NCNTs have two strong peaks at 25.8° and 42.9°. There was no obvious diffraction shift after doping with N. Meanwhile, the peak at 9.6° lower than 2H MoS_2_ 14.4° (JCPDS card No. 37-1492) is attributed to the (002) plane of 1T-MoS_2_/NCNTs, 1T-MoS_2_, and 1T-MoS_2_/CNTs, corresponding to the d-spacing of 0.93 nm, which may be attributed to the intercalation of ${\mathrm{NH}}_{4}^{+}$ from urea during the hydrothermal process [25,26,27,28,29]. In addition, there are obviously sharp and broader diffraction peaks among 1T-MoS_2_, 1T-MoS_2_/CNTs, and 1T-MoS_2_/NCNTs, demonstrating the nanoscale of the 1T-MoS_2_ crystallites in all samples. The Raman spectra (Figure 2b) of the as-prepared samples show two characteristic peaks at 378.4 and 401.4 cm^−1^ attributed to the $E_{2g}^{1}$ and A_1g_ vibrational modes attributed to MoS_2_ which can be observed from the as-prepared samples. The presence of the J_1_ (149 cm^−1^), J_2_ (221 cm^−1^), and J_3_ (335 cm^−1^) peaks in the lower frequency region is attributed to the vibrational modes of the 1T-phase MoS_2_ [34,35]. Furthermore, there exist two distinct peaks at 1346 and 1575 cm^−1^ in the CNTs materials and at 1340 and 1569 cm^−1^ in the NCNTs materials, consisting of the D and G bands of carbon, respectively. The peak intensity ratio I_D_/I_G_ in NCNTs (1.2) is higher than that in CNTs (1.14), indicating the presence of more defects and disordered carbon framework after N-doping [30,31,32,36].

**Figure 3:**
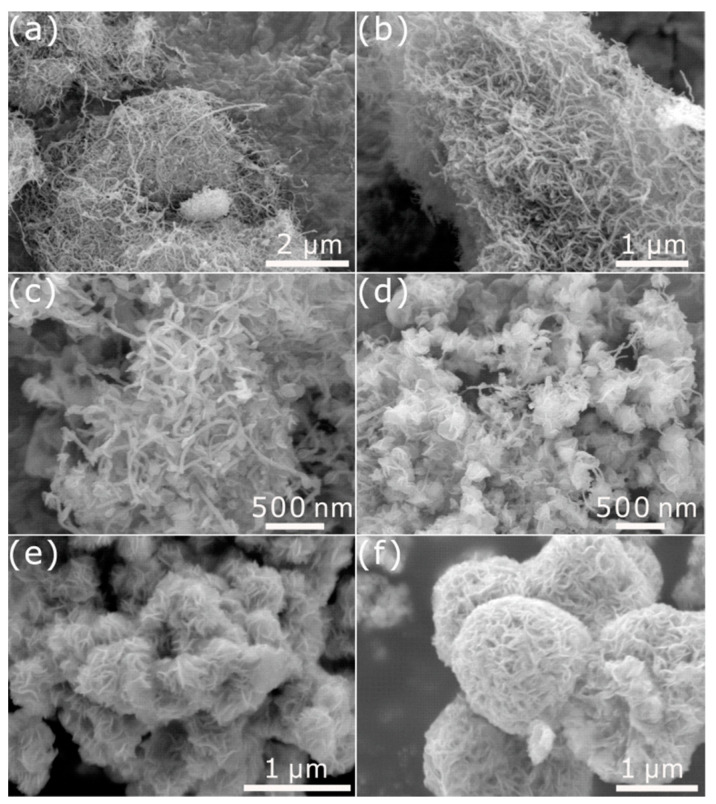
SEM images of (**a**) the CNTs, (**b**) NCNTs, (**c**) 1T-MoS_2_/CNTs, (**d**) 1T-MoS_2_/NCNTs, and (**e**,**f**) 1T-MoS_2_ nanoflowers.

**Figure 4:**
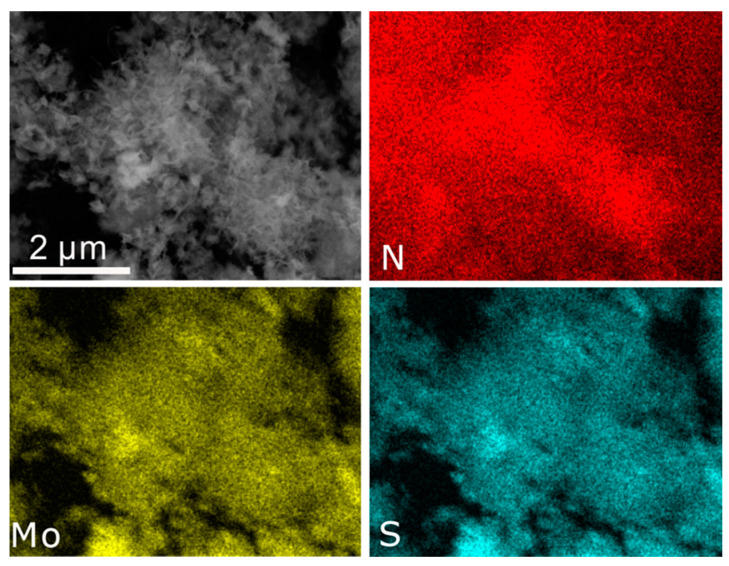
EDS elemental mapping of Mo, S, and N in 1T-MoS_2_/NCNTs.

The morphology of the as-prepared samples was characterized by SEM, as shown in Figure 3. Figure 3a,b show the SEM images of the CNTs and NCNTs. CNTs distributed in a random direction. After the CNTs were doped with N, the morphology of the NCNTs was similar to that of CNTs. Additionally, it can be observed that 1T-MoS_2_ nanosheets distributed on the CNTs without clear agglomeration (Figure 3c). Meanwhile, the multilayer 1T-MoS_2_ nanosheets assembled on the CNTs well. It is worth noting that the number of 1T-MoS_2_ nanosheets assembled on the NCNTs is apparently higher than that on the CNTs (Figure 3d), which may indicate improved anchoring of the 1T-MoS_2_ nanosheets on CNTs after N-doping. Additionally, the 1T-MoS_2_ nanosheets growing uniformly on the NCNTs could provide great electronic conductivity between 1T-MoS_2_ nanosheets and NCNTs and could also expose numerous active sites, promoting HER performance. What is more, the 1T-MoS_2_ nanosheets in nanoflowers (Figure 3e–f) agglomerated together, which thwarted the exposure of active sites on the basal plane and edges. Moreover, the EDS elemental mapping images shown in Figure 4 indicate the existence of N, and it can be observed that the elements Mo and S in the 1T-MoS_2_ nanosheets are distributed uniformly.

**Figure 5:**
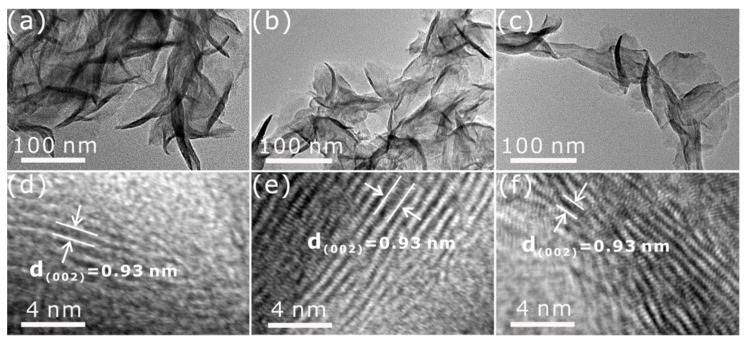
TEM images of (**a**,**d**) 1T-MoS_2_ nanoflowers, (**b**,**e**) 1T-MoS_2_/NCNTs, and (**c**,**f**) 1T-MoS_2_/CNTs.

TEM images (Figure 5a,d) show that the 1T-MoS_2_ nanoflowers have a chaotic nanosheet arrangement, and the lattice spacing is 0.93 nm, corresponding to the (002) lattice plane. Furthermore, it is worth noting that the TEM images (Figure 5b,e) of the as-prepared 1T-MoS_2_ nanosheets anchored on NCNTs show a large lattice spacing of 0.93 nm ascribed to the (002) plane, similar to the lattice spacing observed in the 1T-MoS_2_/CNTs (Figure 5c,f). The large lattice spacing may be attributed to the intercalation of ${\mathrm{NH}}_{4}^{+}$ from urea during the hydrothermal process [37,38].

**Figure 6:**
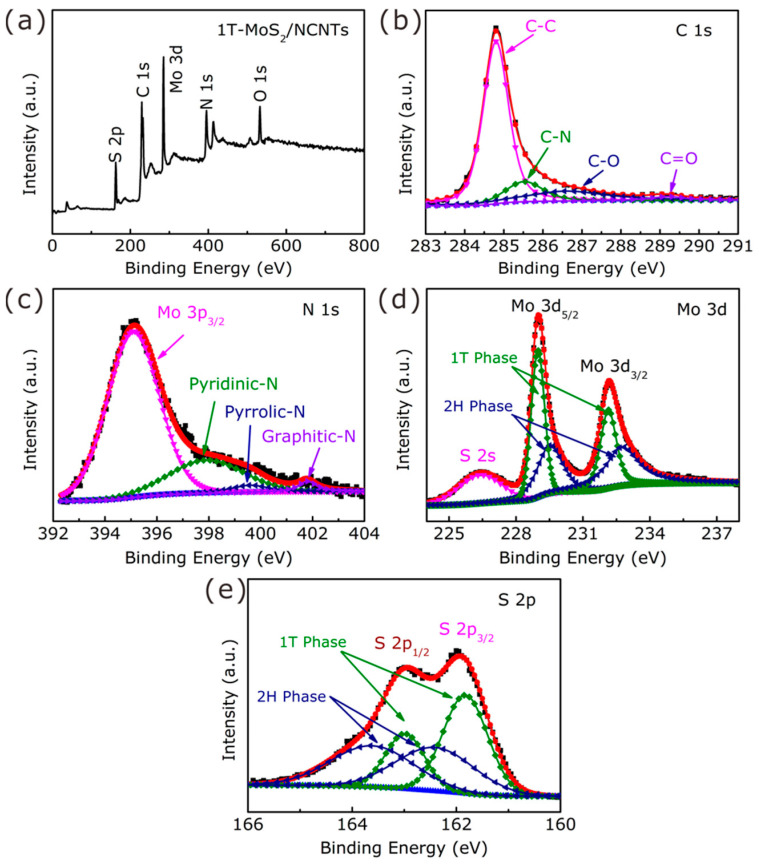
Full-scan XPS spectrum (**a**) and C 1s (**b**), N 1s (**c**), Mo 3d (**d**), and S 2p (**e**) core-level spectra of the 1T-MoS_2_/NCNTs.

The chemical composition and valence states of the 1T-MoS_2_/NCNTs were characterized by XPS. The full-scan XPS spectrum of the 1T-MoS_2_/NCNTs (Figure 6a) shows the existence of the elements C, N, O, Mo, and S. The C 1s core-level spectrum exhibits four peaks located at 284.8, 285.5, 286.6, and 289.2 eV corresponding to C–C, C–N, C–O, and C=O bonds, respectively. The presence of C–O and C=O bonds may refer to the oxygen absorbed from the air [34,39]. The N 1s core-level spectrum of 1T-MoS_2_/NCNTs (Figure 6c) indicates the existence of pyridinic N (397.9 eV), pyrrolic N (399.6 eV), and graphic N (401.8 eV). The strong pyridinic N (397.9 eV) can confirm the N-doping in the carbon nanotubes. In addition, the dopant N atoms prefer to increase the electron density around C atoms, enhance the electrochemical conductivity, and enhance the HER performance [39,40]. Figure 6d illustrates the Mo 3d spectrum of 1T-MoS_2_/NCNTs. The peaks at around 228.9 and 232.1 eV correspond with the binding energy of Mo^4+^ 3d_5/2_ and 3d_3/2_ in 1T-MoS_2_, while the other two peaks located at 229.5 and 232.7 eV can be attributed to Mo^4+^ 3d_5/2_ and 3d_3/2_ in 2H-MoS_2_. Furthermore, the content of 1T-MoS_2_ was about 50% in the 1T-MoS_2_/NCNTs. Likewise, the 1T-phase content can also be obtained through the S 2p core-level spectrum (Figure 6e). It is worth noting that the 1T phase fabrication would likely rely on the intercalation of ${\mathrm{NH}}_{4}^{+}$ ions from urea, and the specific intercalation process is as follows. Firstly, ${\mathrm{NH}}_{4}^{+}$ ions prefer to adsorb on the outer MoS_2_ crystallite surfaces, and then, the weakening of van der Waals forces can provide convenience for diffusion into the next interior layer of the ${\mathrm{NH}}_{4}^{+}$ ions. After ${\mathrm{NH}}_{4}^{+}$ ion intercalation, it can enlarge the interlayer spacing, causing the changing of the crystal structure from 2H to 1T and charge transfer from the intercalator to the host materials, which can not only improve the material’s conductivity but also facilitate electron transfer at the interface between the catalyst and electrolytes, enhancing the HER performance [41,42].

**Figure 7:**
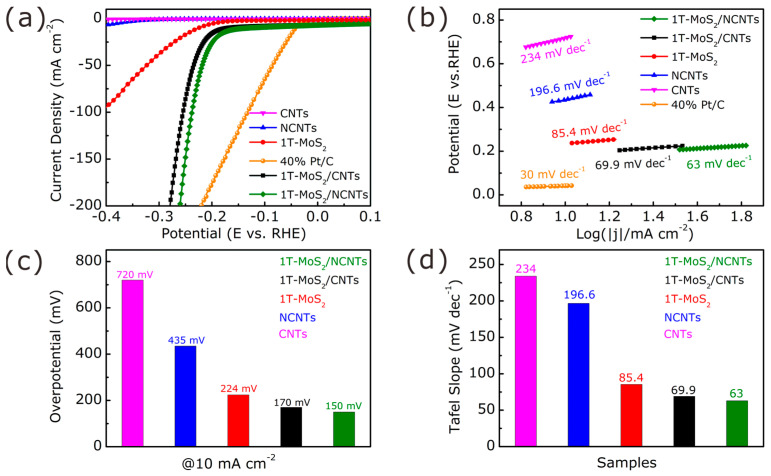
Comparison of the HER performance of NCNTs anchored by the 1T/2H mixed-phase MoS_2_ nanosheets. (**a**) LSV curves. (**b**) Tafel slope curves. (**c**) Comparison of overpotential at 10 mA cm^−2^. (**d**) Comparison of Tafel slopes.

To investigate the influence of N-doped carbon nanotubes on the catalytic activity, the HER performances of 1T-MoS_2_/NCNTs, 1T-MoS_2_/CNTs, 1T-MoS_2_, CNTs, and NCNTs were evaluated in an Ar-saturated 0.5 M H_2_SO_4_ solution. As shown in Figure 7a, a superior HER performance was shown by the commercial Pt/C catalyst, possessing low onset potential and large current density. Apart from the commercial Pt/C catalyst, the 1T-MoS_2_/NCNTs catalyst also exhibited a superior catalytic performance compared with the 1T-MoS_2_/CNTs catalyst, with a distinct difference between the 1T-MoS_2_/NCNTs and 1T-MoS_2_/CNTs catalysts in overpotential at 10 mA cm^−2^, onset potential, and large current density. However, the composites of 1T-MoS_2_ anchored in CNTs and NCNTs revealed better catalytic performances than the 1T-MoS_2_ catalyst, demonstrating the synergistic effect between 1T-MoS_2_ and CNTs with great electrical conductivity, which plays a significant role in enhancing electrochemical catalytic performance. This result can be explained by the fact that 1T-MoS_2_ not combined with a conductive base has poor conductivity for electron transfer from the interior to active sites, and 1T-MoS_2_ nanoflowers’ severe aggregation limits the exposure of more reactive sites. A fantastic phenomenon can be observed whereby N-doped carbon nanotubes show better electrochemical catalytic performance than carbon nanotubes resulting from the electron density changing with N atoms doping.

The superior HER performance of the 1T-MoS_2_/NCNTs catalyst becomes more prominent through the comparison of the Tafel slopes (Figure 7b). The smaller the Tafel slope value is, the larger the current density is at the same potential value. One can see that the 1T-MoS_2_/NCNTs catalyst exhibits a much lower Tafel slope value (63 mV dec^−1^) compared with the 1T-MoS_2_/CNTs (69.9 mV dec^−1^) and 1T-MoS_2_ catalysts (85.4 mV dec^−1^). The kinetic models of HER in acidic conditions can explain that different Tafel slope values are controlled by different rate-determining steps during the HER process, which means that when the value equals 120 mV dec^−1^, the Volmer step (H^+^ + e^−^→H_ads_) reacts as a rate-determining step; when the value is equal to 40 mV dec^−1^, the Heyrovsky step (H^+^ + e^−^ + H_ads_→H_2_) reacts as a rate-determining step; and when the value equals 30 mV dec^−1^, the Tafel (H_ad_ + H_ads_→H_2_) step reacts as a rate-determining step [43]. The small Tafel slope of 63 mV dec^−1^ indicates that the kinetic model of 1T-MoS_2_/NCNTs is the Volmer-Heyrovsky mechanism in the HER process, and electrochemical desorption reacts as the rate-limiting step, which can also explain the kinetic model of 1T-MoS_2_/CNTs and 1T-MoS_2_. Moreover, the samples of CNTs and NCNTs have large Tafel slopes of 234 and 196.6 mV dec^−1^, respectively, belonging to the Volmer-Tafel mechanism. In addition, Figure 7c shows the overpotential at 10 mA cm^−2^ of all the as-prepared catalysts, demonstrating that the 1T-MoS_2_/NCNTs catalyst has a lower overpotential of 150 mV. Meanwhile, the changing value of Tafel slopes (Figure 7d) shows a similar pattern to that of overpotential at 10 mA cm^−2^ (Figure 7c).

**Figure 8:**
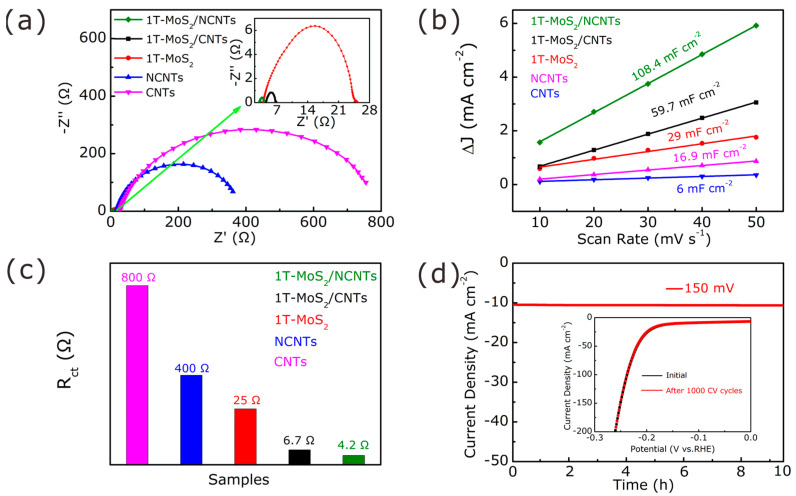
(**a**) EIS plots of as-prepared samples; (**b**) ECSA of 1T-MoS_2_/NCNT, 1T-MoS_2_/CNT, 1T-MoS_2_, CNTs, and NCNTs in 0.5 M H_2_SO_4_; (**c**) comparison of *R_CT_*; (**d**) long-time chronoamperometric test performance at an overpotential of 150 mV. Inset illustrates initial LSV compared to curve after 1000 cycles.

To better understand the electrochemical mechanism, EIS was applied to measure the transfer resistance (Figure 8a). The charge transfer resistance (*R_CT_*) of the as-prepared electrochemical catalysts increased remarkably from 4 to 800 Ω (Figure 8c), which can be ascribed to the decrease in active sites and electrical conductivity. One can see that the 1T-MoS_2_/NCNTs catalyst possesses a lower *R_CT_* value of around 4.2 Ω ascribed to the synergistic effect between 1T-MoS_2_ nanosheets exposed as much as possible to active sites and N-doped carbon nanotubes possessing great electrical conductivity, enhancing the composites’ HER performance drastically. What is more, the smaller the value of *R_CT_* is, the better the electron transfer is and the easier the electrode kinetics are, thus boosting the HER performance.

**Figure 9:**
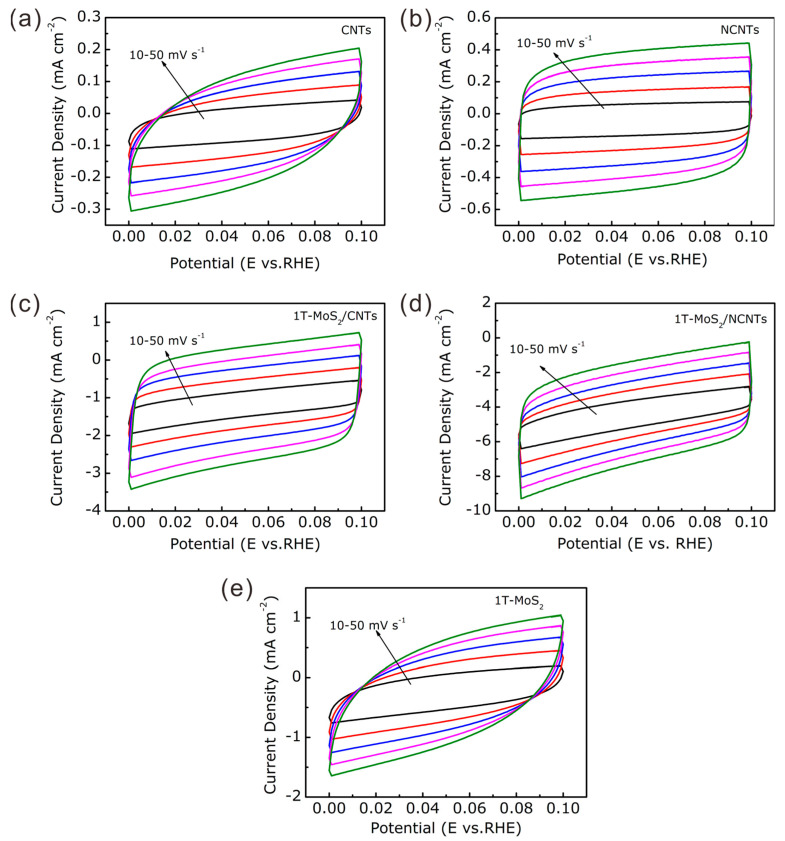
CV curves at different scan rates from 10 to 50 mV s^−1^ of (**a**) CNTs, (**b**) NCNTs, (**c**) 1T-MoS2/CNTs, (**d**) 1T-MoS2/NCNTs, and (**e**) 1T-MoS2 nanoflowers.

The *C*_dll_ is another important tool for electrocatalysts to reflect the electrochemical active surface areas (ECSAs), which can be derived from the cyclic voltammetry (CV) curves at different scan rates (Figure 9). As shown in Figure 8b, the *C*_dll_ of the 1T-MoS_2_/NCNTs catalyst was 108.4 mF cm^−2^, which is the largest among all of the as-prepared catalysts. This result could be attributed to the synergistic effect between 1T-MoS_2_ and NCNTs; the former provides as great an active surface as possible, and the latter possesses great electrical conductivity. The large active surface possessing plentiful active sites prefers to access reactants in the electrolyte easily, further promoting the H adsorption/desorption behavior during the HER process.

**Figure 10:**
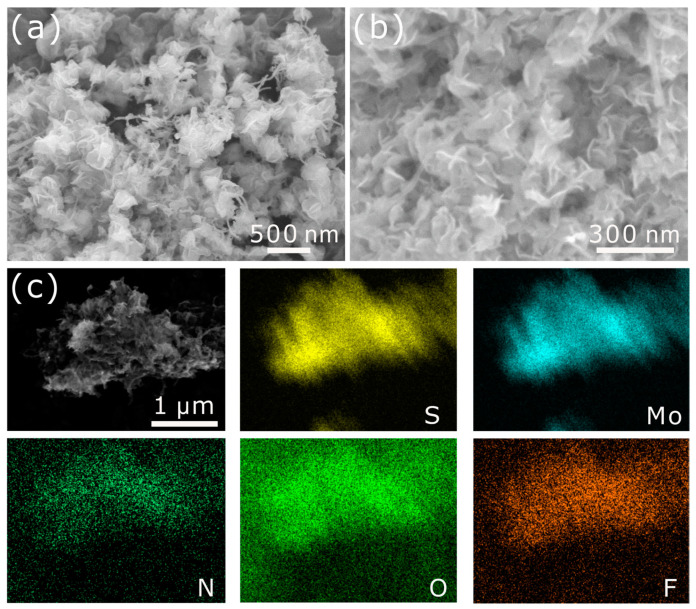
(**a**) SEM pattern of 1T-MoS_2_/NCNTs catalyst before HER; (**b**) SEM pattern of 1T-MoS_2_/NCNTs catalyst after HER; (**c**) EDS elemental mapping of Mo, S, N, O, and F in 1T-MoS_2_/NCNTs after HER.

**Figure 11:**
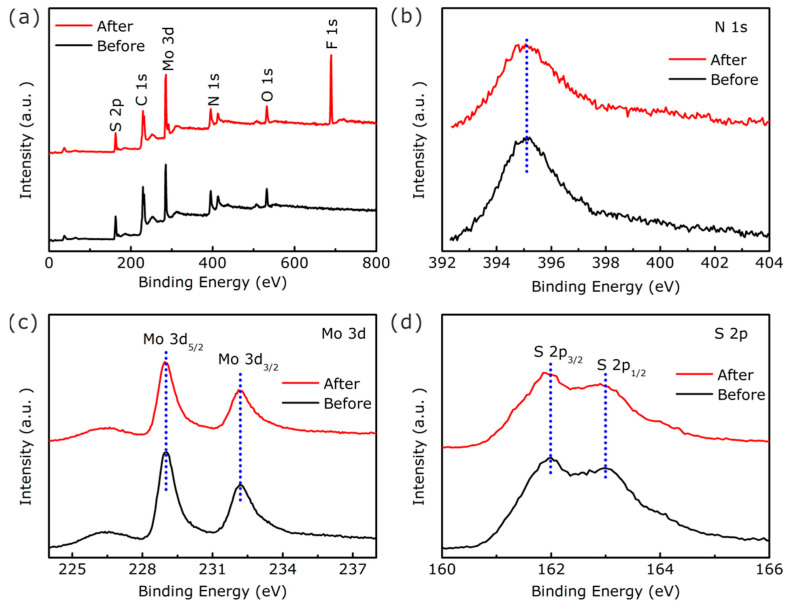
Full-scan XPS spectra (**a**) and N 1s (**b**), Mo 3d (**c**), and S 2p (**d**) core-level spectra of fresh and spent electrocatalysts.

Electrochemical stability is another significant evaluation index for electrocatalysts. The stability evaluation of the 1T-MoS_2_/NCNTs catalyst was evaluated and is shown in Figure 8d. The current density of the 1T-MoS_2_/NCNTs catalyst showed no obvious activity degradation after 10 h testing at a constant overpotential of 150 mV, demonstrating great stability in 0.5 M H_2_SO_4_ solution. Meanwhile, a long-term cycling test was performed using CV for 1000 cycles. As shown in the Figure 8d inset, no distinct activity loss can be seen from the LSV curve after 1000 cycles compared to the initial LSV curve. To further confirm the great electrochemical stability of the 1T-MoS_2_/NCNTs catalyst, SEM and XPS analyses were applied to investigate the electrochemical stability (Figure 10 and Figure 11). Figure 10a,b show the SEM patterns of the 1T-MoS_2_/NCNTs catalyst before and after HER. From the SEM patterns, it can be seen that the morphology of the 1T-MoS_2_/NCNTs catalyst after HER is similar to that of the catalyst before HER, illustrating that the HER process has negligible effects on the morphology of the 1T-MoS_2_/NCNTs catalyst. The existence of F and O elements is attributed to the Nafion solution (Figure 10c). The comparison of XPS data (Figure 11) before and after HER of the 1T-MoS_2_/NCNTs catalyst can also confirm the good stability of the as-prepared samples.

## 4. Conclusions

In summary, we designed and prepared 1T-MoS_2_/NCNTs composite by anchoring 1T-MoS_2_ nanosheets on NCNTs. ${\mathrm{NH}}_{4}^{+}$ ions from urea enlarged the interlayer of MoS_2_, forming the 1T phase. The N-doping of carbon nanotubes changed the electron density, improving the conductivity of the carbon nanotubes. Abundant catalytic active sites of 1T-MoS_2_ nanosheets were achieved by regulating the reaction temperature and time. The 1T-MoS_2_ nanosheets were vertically anchored on the N-doped carbon nanotubes. Benefiting from the synergistic effects of these features, the 1T-MoS_2_/NCNTs catalyst exhibited a superior HER performance in 0.5 M H_2_SO_4_, including a lower overpotential (150 mV at 10 mA cm^−2^), a smaller Tafel slope (63 mV dec^−1^), and remarkable electrochemical stability. This work thus proposes a new direction for designing high-performance HER catalysts.

## Data Availability

The datasets generated during and/or analyzed during the current study are available from the corresponding author.
